# Supplementary material for: Identifying developmental trajectories of body mass index in childhood using latent class growth (mixture) modelling: associations with dietary, sedentary and physical activity behaviors: a longitudinal study
Source: BMC Public Health. 2016 Oct 28;16:1128. doi: 10.1186/s12889-016-3757-7 (PMC5086035; doi:10.1186/s12889-016-3757-7)
Supplement: Additional file 1: Table S1. — Characteristics of the study population based on the three-class model. Table S2. Health behaviors in the BMI SDS trajectories: the three-class model (DOC 95 kb) [file 12889_2016_3757_MOESM1_ESM.doc]

Table S1. Characteristics of the study population based on the three-class model

| **Variables of interest**  **[%] or [mean (SD)]** | **BMI trajectories** | | |  | | **P-value ͣ** | |
| --- | --- | --- | --- | --- | --- | --- | --- |
|  | **Stable high**  **(n= 212)** | **Decreasing (n=346)** | **Steeply increasing (n=55)** | | **Total sample**  **(N = 613)** | |  |
| Gender % male | 56.6 | 45.7 | 54.5 | | 50.2 | |  |
| SES |  |  |  | |  | |  |
| low | 15.6 | 9.2 | 18.2 | | 12.1 | | ** |
| middle | 25.9 | 21.1 | 23.6 | | 22.3 | | ** |
| high | 58.5 | 69.7 | 58.2 | | 65.6 | | ** |
| Ethnicity – % non-western | 11.8 | 6.1 | 10.9 | | 8.5 | | ** |
| BMI |  |  |  | |  | |  |
| 2006 | 16.4 (1.4) | 15.3 (1.1) | 14.6 (1.0) | | 15.6 (1.33) | | ** |
| 2009 | 17.3 (2.2) | 15.0 (1.3) | 16.0 (1.7) | | 16.0 (2.0) | | ** |
| 2012 | 19.6 (2.3) | 16.1 (1.2) | 19.4 (2.7) | | 17.6 (2.5) | | ** |
| Waist circumference |  |  |  | |  | |  |
| 2006 | 54.8 (4.1) | 52.8 (6.0) | 51.3 (3.6) | | 53.4 (5.4) | | ** |
| 2009 | 61.7 (6.9) | 56.5 (3.4) | 60.6 (4.6) | | 58.7 (5.5) | | ** |
| 2012 | 71.0 (7.5) | 61.7 (4.1) | 71.4 (8.3) | | 65.7 (7.5) | | ** |
| **Weight status child (%)** |  |  |  | |  | |  |
| Thinness |  |  |  | |  | |  |
| 2006 | 1.9 | 12.4 | 30.9 | | 10.4 | | ** |
| 2009 | 0.8 | 21.3 | 16.2 | | 13.8 | | ** |
| 2012 | 0.0 | 18.8 | 0.0 | | 10.6 | | ** |
| Healthy weight |  |  |  | |  | |  |
| 2006 | 82.1 | 84.7 | 69.1 | | 82.4 | | ** |
| 2009 | 77.8 | 77.3 | 75.7 | | 77.3 | | ** |
| 2012 | 77.8 | 81.2 | 76.4 | | 79.6 | | ** |
| Overweight (including obesity) |  |  |  | |  | |  |
| 2006 | 16.1 | 2.9 | 0.0 | | 7.2 | | ** |
| 2009 | 21.5 | 1.4 | 8.1 | | 7.3 | | ** |
| 2012 | 22.2 | 0.0 | 23.6 | | 8.3 | | ** |
| **Weight status parent** |  |  |  | |  | |  |
| BMI mother (% overweight) |  |  |  | |  | |  |
| 2006 | 39.3 | 21.0 | 46.6 | | 29.6 | | ** |
| 2012 | 37.4 | 24.8 | 36.7 | | 30.2 | | ** |
| BMI father (% overweight) |  |  |  | |  | |  |
| 2006 | 54.8 | 44.2 | 48.7 | | 48.1 | |  |
| 2012 | 55.8 | 44.4 | 56.3 | | 49.4 | | ** |

Abbreviations: BMI, body mass index (calculated as weight in kilograms divided by height in metres squared); SDS, standard deviation scores; SES, socioeconomic status; SD, standard deviation

ᵃ P-values determined using X² test (categorical variables) or Anova (continuous variables) * p<0.05 for difference between trajectories; ** p<0.001 for difference between trajectories

Table S2. Health behaviors in the BMI SDS trajectories: the three-class model

| **Variables of interest [%]** | **BMI trajectories** | | | |  | **P-value ͣ** | |
| --- | --- | --- | --- | --- | --- | --- | --- |
|  | **Stable high**  **(n= 212)** | **Decreasing (n=346)** | **Steeply increasing (n=55)** | **Total sample**  **(N = 613)** | | |  |
| **Dietary behaviors** |  |  |  |  | | |  |
| Vegetable intake < 5 days a week |  |  |  |  | | |  |
| 2006 | 58.7 | 55.7 | 60.0 | 57.1 | | |  |
| 2012 | 57.3 | 58.4 | 53.6 | 57.4 | | |  |
| Fruit < 2 portions a day |  |  |  |  | | |  |
| 2006 | 69.9 | 74.4 †ᵇ | 73.8 | 72.8 †† | | |  |
| 2012 | 76.0 | 83.5 † | 77.8 | 80.4 †† | | | ** |
| Sugared drinks > 3 glasses a day |  |  |  |  | | |  |
| 2006 | 55.6 †† | 44.5 † | 47.1 | 48.6 †† | | | * |
| 2012 | 38.5 †† | 34.2 † | 36.1 | 35.9 †† | | |  |
| **Physical activity behaviors** |  |  |  |  | | |  |
| Outside play < 1 hour a day |  |  |  |  | | |  |
| 2006 | 62.7 † | 57.4 | 41.9 | 57.8 † | | | * |
| 2012 | 50.5 † | 51.5 | 49.1 | 50.6 † | | |  |
| Organized sports < 1 hour a week |  |  |  |  | | |  |
| 2006 | 67.0 † | 68.7 †† | 59.6 † | 67.3 †† | | |  |
| 2012 | 20.5 † | 15.5 †† | 14.0 † | 16.7 †† | | | * |
| **Sedentary behaviors** |  |  |  |  | | |  |
| TV viewing > 2 hours a day |  |  |  |  | | |  |
| 2006 | 3.4% †† | 2.7% †† | 6.0% | 3.2 †† | | |  |
| 2012 | 14.1% †† | 9.4% †† | 14.8% | 11.5 †† | | |  |
| Screentime > 2 hours a day |  |  |  |  | | |  |
| 2006 | 6.5 †† | 5.3 †† | 6.1 †† | 5.8 †† | | |  |
| 2012 | 65.6 †† | 53.8 †† | 73.3 †† | 59.7 †† | | | ** |

ᵃ P-values determined using X² test: * p<0.05 for difference between trajectories;

ᵇ P-values determined using X² test : † p<0.05 for differences in behaviors between baseline and follow-up; †† p <0.001 for differences in behaviors between baseline and follow-up
