# Supplementary figures and images for: Identifying developmental trajectories of body mass index in childhood using latent class growth (mixture) modelling: associations with dietary, sedentary and physical activity behaviors: a longitudinal study
Source: BMC Public Health. 2016 Oct 28;16:1128. doi: 10.1186/s12889-016-3757-7 (PMC5086035; doi:10.1186/s12889-016-3757-7)

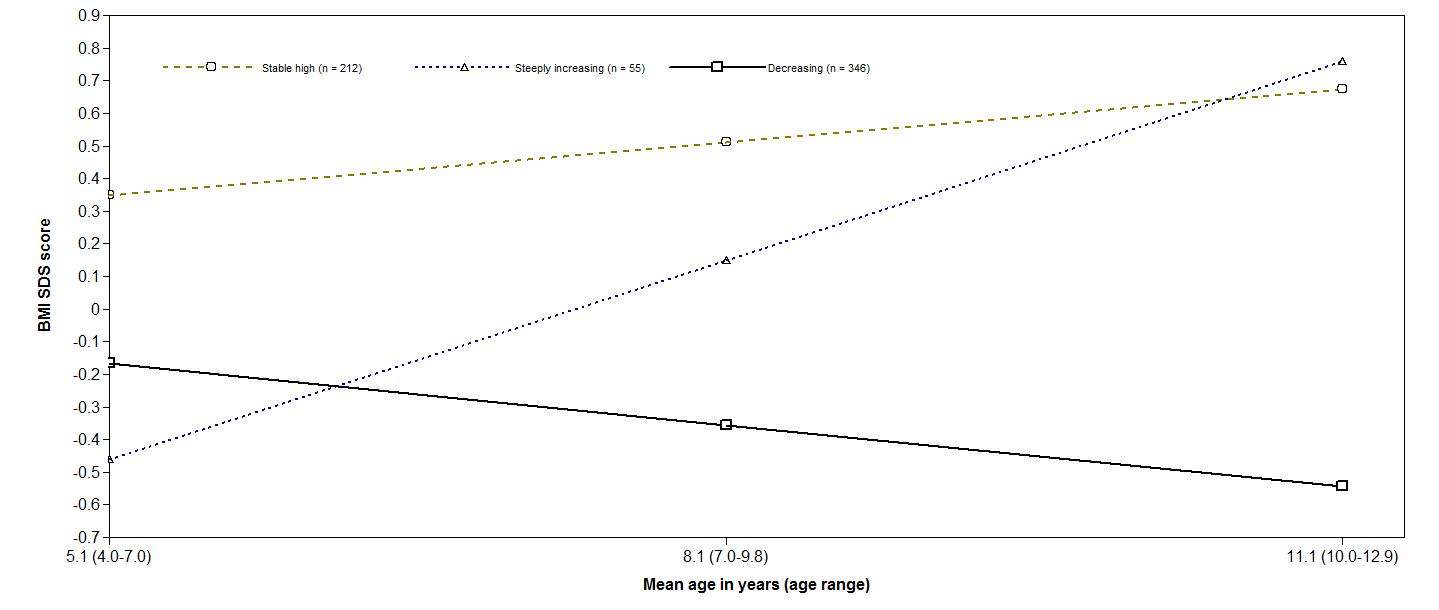

Supplement: Additional file 2: Figure S1. — Three class linear model of BMI SDS trajectories. The latent growth patterns of BMI SDS are represented by mean trajectories of BMI standard deviation scores (SDS) (y-axis) at the different mean ages (x-axis). (JPG 54 kb) [file 12889_2016_3757_MOESM2_ESM.jpg]
